# Influence of Chloride Ions on Corrosion Mechanism of Pipeline Steel in Bicarbonate Alkaline Environments

**DOI:** 10.3390/ma19143112

**Published:** 2026-07-20

**Authors:** Daoyu Liu, Di Jiang, Xinzhu Li, Kunxiang Ge, Peng Cai, Yixuan Huang, Hao Zhang

**Affiliations:** 1 Shanghai Marine Equipment Research Institute, Shanghai 200031, China; 2 Beijing Advanced Innovation Center for Materials Genome Engineering, Institute for Advanced Materials and Technology, University of Science and Technology Beijing, Beijing 100083, China

**Keywords:** pipeline steel, corrosion mechanism, chloride ion, alkaline electrolyte

## Abstract

The corrosion evolution of pipeline steel was investigated in NaCl + 0.75 M NaHCO_3_ solutions with different chloride concentrations using potentiodynamic polarization, electrochemical impedance spectroscopy (EIS), Mott–Schottky analysis, X-ray photoelectron spectroscopy (XPS), and surface characterization techniques. The results demonstrated that chloride concentration was a critical factor governing the transition of corrosion behavior from a relatively stable oxide-film-controlled state to active dissolution. At low Cl^−^ concentrations, a stable current-density region was observed, indicating the formation of a protective oxide film. With increasing Cl^−^ concentration, the stable current region gradually disappeared, accompanied by a continuous decrease in polarization resistance and oxide-film resistance, revealing the progressive deterioration of corrosion resistance. Mott–Schottky analysis showed that the oxide film formed in 0.5 wt.% NaCl + 0.75 M NaHCO_3_ solution (pH ≈ 8.3) exhibited n-type semiconductor characteristics, with a donor density of 8.69 × 10^21^ cm^−3^. XPS analysis revealed that the oxide film mainly consisted of Fe^2+^-related oxides. The experimental results indicate that the corrosion evolution was associated with the competitive interaction between ${HCO}_{3}^{-}$ and Cl^−^, where bicarbonate favored oxide-film stabilization at low chloride concentrations, whereas chloride enrichment promoted oxide-film deterioration and accelerated active dissolution. This study establishes the relationship between chloride concentration variation, oxide-film degradation, and corrosion evolution of pipeline steel in high-bicarbonate alkaline environments, providing experimental insights for corrosion assessment and control under chloride-enriched service conditions.

## 1. Introduction

Carbon steel due to its low cost, excellent mechanical properties, and good processability remains one of the most widely used structural materials in pipeline systems for oil and gas transportation, chemical processing, and marine engineering applications [1,2]. However, during long-term service, pipeline steels are inevitably exposed to complex corrosive environments, and corrosion failures may significantly reduce service life and lead to leakage, environmental pollution, and safety hazards [3]. Therefore, understanding corrosion behavior and mechanisms under realistic service conditions is of great importance.

In industrial systems such as petroleum extraction, oil and gas transportation, and chlor-alkali brine purification, bicarbonate ions (${HCO}_{3}^{-}$) are widely present in service environments [4]. ${HCO}_{3}^{-}$ participates in ion regulation and is often used as a buffering agent to control corrosion within acceptable limits [4,5]. On the other hand, chloride ions (Cl^−^), as typical aggressive anions, are widely present in industrial environments and often coexist with ${HCO}_{3}^{-}$ [6]. The Cl^−^ concentration can vary significantly depending on service conditions, and in localized regions such as crevices and corrosion product-covered areas, chloride enrichment may occur due to restricted mass transport [7]. Therefore, chloride concentration variation is a key factor influencing corrosion behavior. The chloride concentration may vary over a wide range (approximately 0.5–20 wt.%) depending on environmental conditions [4]. For example, seawater contains about 3.5 wt.% NaCl, while in confined regions such as under-deposit zones, chloride ions may become highly concentrated due to local accumulation effects [7,8].

Extensive studies have investigated the effects of ${HCO}_{3}^{-}$ and Cl^−^ on pipeline steel corrosion. ${HCO}_{3}^{-}$ has been reported to influence corrosion processes and induce transitions between active dissolution and passivation states [9]. It may also act as an additional proton source, accelerating corrosion reactions [10]. Moreover, ${HCO}_{3}^{-}$ can compete with chloride ions for adsorption sites on steel surfaces, thereby reducing corrosion activity [11]. At higher concentrations, ${HCO}_{3}^{-}$ promotes the formation of Fe (II)/Fe (III)-based oxide films, improving film compactness and stability and reducing corrosion rates [12,13]. In contrast, Cl^−^ tends to preferentially adsorb on the oxide film surface and penetrate through the oxide film to the metal/film interface, inducing defect formation and localized dissolution [13,14]. This weakens the protective ability of the oxide film and promotes localized corrosion [14,15]. In ${HCO}_{3}^{-}$/Cl^−^ coexisting systems, competitive adsorption and synergistic interactions jointly influence film formation and electrochemical behavior, indicating that corrosion behavior depends on their interaction rather than a single ion effect [11,16].

However, in real pipeline service environments, Cl^−^ concentration is not constant but dynamically varies due to crevice effects, deposits, and local ion accumulation [8]. Therefore, chloride concentration variation represents an important environmental parameter influencing corrosion behavior. In high-${HCO}_{3}^{-}$ alkaline environments, the interfacial chemistry of pipeline steel is strongly buffered, facilitating the formation of a relatively stable oxide film prior to chloride exposure. Under such conditions, progressive chloride enrichment may alter oxide film stability, defect characteristics, and the subsequent corrosion evolution. Although the effects of Cl^−^ on steel corrosion have been widely investigated, previous studies have mainly focused on the presence of Cl^−^ or fixed Cl^−^ concentrations. The influence of continuously varying Cl^−^ concentrations on the evolution of oxide-film properties and the transition of corrosion behavior under high-bicarbonate alkaline conditions remains insufficiently understood. This knowledge gap is particularly important for understanding the transition from an oxide-film-controlled relatively stable state to an active corrosion state induced by Cl^−^ enrichment.

Based on this consideration, this study investigates the corrosion evolution of pipeline steel in 0.75 M NaHCO_3_ solutions with different Cl^−^ concentrations, aiming to clarify the role of Cl^−^ concentration variation under high-bicarbonate alkaline conditions. Electrochemical measurements, including potentiodynamic polarization, electrochemical impedance spectroscopy (EIS), and Mott–Schottky analysis, were employed to characterize the evolution of oxide-film properties, while surface morphology and corrosion product analyses were conducted to correlate interfacial changes with corrosion behavior. This work is to experimentally establish the relationship between Cl^−^ concentration variation, oxide-film stability, semiconductor characteristics, and corrosion evolution of pipeline steel, providing mechanistic insights and experimental guidance for corrosion control in chloride-enriched alkaline service environments.

## 2. Materials and Methods

### 2.1. Materials and Solutions

**Figure 1:**
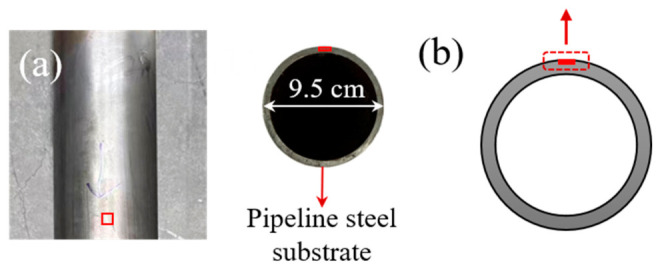
Experimental materials and sampling schematic (the red square indicates the sampling region): (**a**) the pipeline steel; (**b**) sampling schematic.

The pipeline steel used in this study was manufactured from 20 steel, and its chemical composition is listed in Table 1. A schematic illustration of the sampling procedure is shown in Figure 1. All specimens were machined into square coupons with dimensions of 10 mm × 10 mm × 3 mm. Before electrochemical testing, the exposed surfaces were sequentially ground using SiC abrasive papers up to 2000 grit to obtain a uniform surface finish. The specimens were then degreased in ethanol, thoroughly rinsed with deionized water, and dried in cold air before testing.

To simulate different service environments, two groups of electrolytes were prepared: NaCl solutions with concentrations of 0.5 wt.%, 3.5 wt.%, and 5.5 wt.%, and corresponding solutions containing the same NaCl concentrations supplemented with 0.75 M NaHCO_3_. During the electrochemical measurements, the pH of the NaCl solutions was maintained at a neutral level, whereas the pH of the NaCl + NaHCO_3_ solutions was approximately 8.3. All test solutions were freshly prepared using ultrapure water before each experiment to ensure the reproducibility of the results.

### 2.2. Surface Morphology Analysis

A 4% HNO_3_ alcohol solution was used to etch the polished specimens. Subsequently, the specimen surfaces were rinsed with deionized water and ethanol, dried with air, and examined using a ZEISS AxioCam MRc5 optical microscope (Carl Zeiss AG, Oberkochen, Germany) and a ZEISS crossbeam 350 scanning electron microscope (Carl Zeiss AG, Oberkochen, Germany). The particle size was statistically measured using the ImageJ software (v1.54m). The three-dimensional surface morphology and corrosion depth of the specimens after electrochemical polarization tests were characterized using a scanning laser confocal microscope, Keyence VK-X250 (Keyence Corporation, Osaka, Japan). A Bruker MultiMode 8 Scanning Kelvin Probe Force Microscope (SKPFM) (Bruker Corporation, Billerica, MA, USA) was employed to analyze the microstructure morphology and potential distribution.

### 2.3. Electrochemical Tests

All electrochemical measurements were conducted in an aerated solution at room temperature under quiescent conditions. Electrochemical measurements were carried out by CS310 electrochemical workstation (CorrTest Instrument Co., Ltd., Wuhan, China) using a conventional three-electrode electrochemical cell, where the pipeline steel specimen, with an exposed area of 1 cm^2^, served as the working electrode, a platinum sheet acted as the counter electrode, and a saturated calomel electrode (SCE) was used as the reference electrode. All potentials reported in this work are referenced to the SCE. Before each measurement, the working electrode was cathodically polarized at −1.3 V_SCE_ for 3 min to remove the air-formed oxide film from the surface. Subsequently, the open circuit potential (OCP) was monitored for 0.5 h. After the OCP reached a relatively stable value, electrochemical impedance spectroscopy (EIS) and potentiodynamic polarization tests were conducted. EIS measurements were performed at the stabilized OCP over a frequency range from 100 kHz to 0.01 Hz with an AC perturbation amplitude of 5 mV. The initial potential was the OCP. The impedance data were analyzed using appropriate equivalent circuit models implemented in the ZSimpWin software (v3.6). Potentiodynamic polarization curves were recorded from −1.0 V_SCE_ to 0.5 V_SCE_ at a scan rate of 0.5 mV s^−1^. Each experiment was repeated at least three times under identical conditions to ensure reproducibility.

For Mott–Schottky analysis, according to the potentiodynamic polarization results, the surface oxide layer was formed potentiostatically at −0.4 V_SCE_ for 1 h. Subsequently, measurements were performed over a potential range from −0.4 to −0.1 V_SCE_ with a potential step of 20 mV and a fixed frequency of 1 kHz. All measurements were repeated multiple times under the same conditions to ensure reproducibility. Each experiment was repeated at least three times under identical conditions to ensure reproducibility.

### 2.4. X-Ray Photoelectron Spectroscopy

The chemical composition of the oxide film on specimens after potentiostatic polarization in the 0.5 wt.% NaCl + NaHCO_3_ solution for 1 h was examined by XPS using an ESCALAB 250Xi (Thermo Fisher Scientific, Waltham, MA, USA), with an Al Kα (hα = 1486.6 eV) operated at 300 W. After polarization, the specimens were rinsed with deionized water, dried, and stored under vacuum before XPS analysis to minimize oxidation during sample handling. The charge shift in the spectra was corrected assuming the C 1s peak was at 284.8 eV.

## 3. Results and Discussion

### 3.1. Microstructural Observation and Analysis

**Figure 2:**
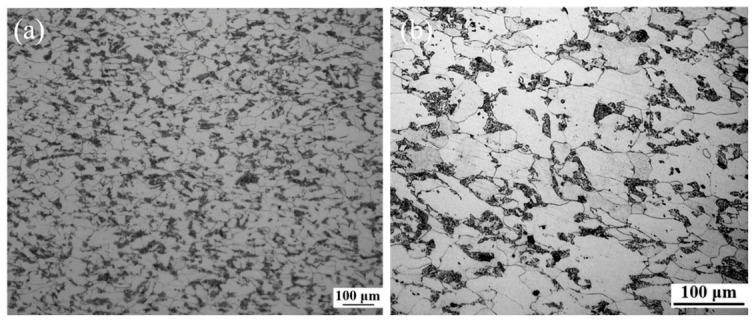
Microstructural observation of pipeline steel: (**a**) 100×; (**b**) 200×.

To characterize the microstructure of the pipeline steel, Figure 2 presents its optical microscopy (OM) images. The microstructure is primarily composed of pearlite and ferrite. The average grain size was statistically analyzed using ImageJ software, and the results indicate that the average grain size of the pipeline steel is approximately 26.48 ± 6.54 μm. In addition, quantitative image analysis was performed, revealing that the pearlite area fraction in the pipeline steel is approximately 24.45%.

**Figure 3:**
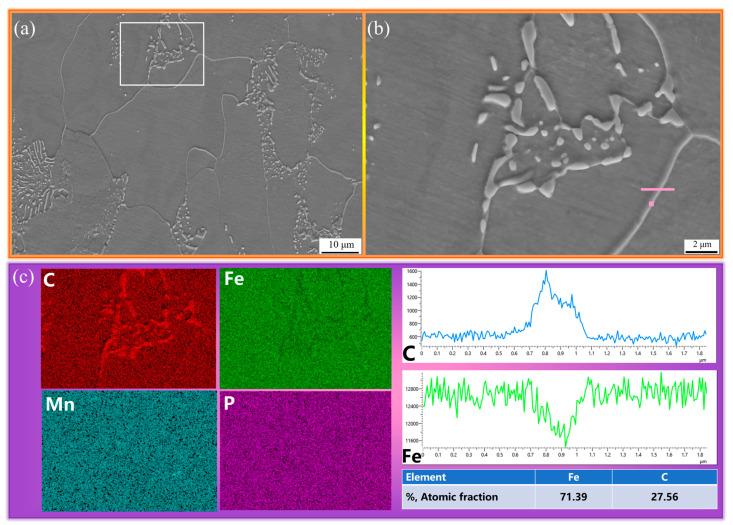
Microstructural analysis of pipeline steel: (**a**) SEM image of the microstructure; (**b**) enlarged detail of the white-boxed region in (**a**); (**c**) EDS mapping, line scan (pink line), and quantitative point analysis (pink dots) of region (**b**).

Figure 3 presents the scanning electron microscopy (SEM) micrographs and elemental distribution of the pipeline steel specimen. It can be observed that the microstructure is mainly composed of ferrite and lamellar pearlite. EDS analysis was performed on the carbide region marked by the white rectangle. Meanwhile, line scan analysis of carbon and iron was conducted along the pink line at the grain boundary, revealing a noticeable concentration gradient of cementite at the grain boundary. Elemental analysis indicates that these carbides are primarily iron-based carbides, with an atomic ratio of iron to carbon of approximately 3:1. Considering the composition of the investigated pipeline steel, these carbides are inferred to be cementite (Fe_3_C).

**Figure 4:**
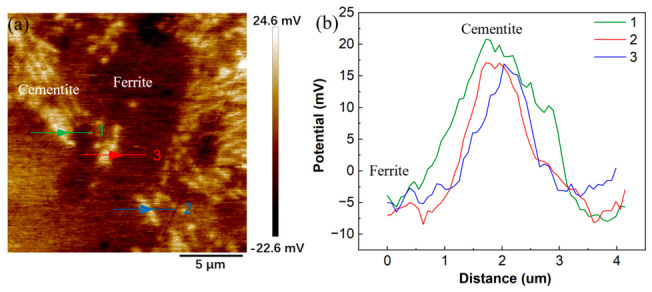
Surface morphology and corresponding SKPFM potential distribution of the pipeline steel: (**a**) SKPFM potential map; (**b**) potential profile along the line marked in (**a**).

The microstructure of the pipeline steel was further characterized. Figure 4 shows the surface morphology and corresponding SKPFM potential distribution of the pipeline steel. In Figure 4a, the dark regions correspond to ferrite, while the light lamellar structures correspond to pearlite, in which ferrite and cementite are arranged in an alternating lamellar configuration. As shown in Figure 4b, a clear potential difference exists between ferrite and pearlite. Specifically, a potential difference is observed between cementite and ferrite within the pearlitic colonies, where cementite acts as the cathode and ferrite acts as the anode. In a corrosive environment, the two phases form micro-galvanic couples, which promote the preferential dissolution of ferrite and accelerate the initiation and propagation of localized corrosion [18,19].

### 3.2. Electrochemical Testing and Corrosion Morphology Analysis

**Figure 5:**
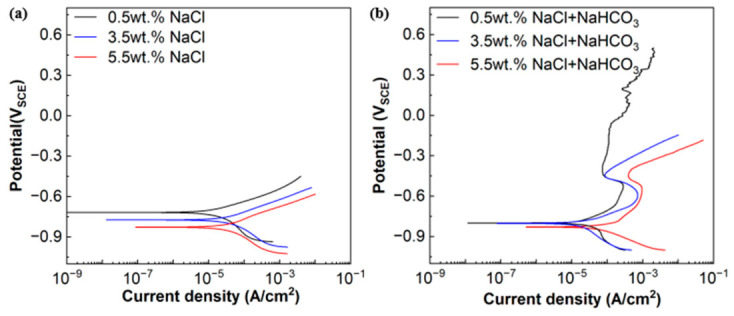
Potentiodynamic polarization curves of pipeline steels in NaCl solutions (**a**) and NaCl + 0.75 M NaHCO_3_ solutions (**b**) with different NaCl concentrations.

Figure 5 presents the potentiodynamic polarization curves of the pipeline steel specimens in different test solutions, while the electrochemical parameters obtained from Tafel linear fitting of the activation region are summarized in Table 2. As shown in the figure, the anodic polarization behavior of the pipeline steel exhibits significant variations with solution composition. In NaCl solutions without the addition of bicarbonate ions (${HCO}_{3}^{-}$), the anodic reaction is entirely governed by activation-controlled dissolution. In the 0.5 wt.% NaCl + NaHCO_3_ solution, an anodic current peak appears at approximately −0.50 V_SCE_, followed by a gradual decrease in anodic current density. Within the potential range from −0.45 to −0.05 V_SCE_, the anodic current density remains nearly constant at approximately 1.05 × 10^−4^ A·cm^−2^. A further increase in anodic current is observed only when the potential exceeds −0.05 V_SCE_, indicating the formation of a protective oxide film on the pipeline steel surface within this potential interval. Similar behavior has been reported for pipeline steels exposed to chloride-free NaHCO_3_ solutions, where one or two anodic current peaks are typically observed before the current enters a stable region [20]. With increasing NaCl concentration, the ${HCO}_{3}^{-}$/Cl^−^ molar ratio in the solution decreases continuously. Correspondingly, the anodic Tafel slope *β_a_* decreases from 0.101 to 0.080 V·dec^−1^, and further to 0.045 V·dec^−1^ in the 5.5 wt.% NaCl + NaHCO_3_ solution, indicating accelerated anodic reaction kinetics. Under these conditions, the anodic process gradually shifts toward active dissolution, and no stable current-density region is observed. Nevertheless, a local decrease in current density still appears near −0.60 V_SCE_, forming an anodic current peak characterized by an initial increase followed by a subsequent decrease in current density. The peak current density increases with increasing NaCl concentration, suggesting that chloride enrichment progressively weakens the stability of the surface film and promotes active anodic dissolution.

As summarized in Table 2, increasing the Cl^−^ concentration results in a negative shift in the corrosion potential (*E_corr_*) and an increase in the corrosion current density (*i_corr_*) for specimens exposed to the same solution environment. This behavior indicates that the higher Cl^−^ concentration enhances the electrical conductivity of the solution, thereby accelerating the corrosion rate of the pipeline steel. A comparison of the results further reveals that, in alkaline chloride-containing environments, the variation in *E_corr_* with Cl^−^ concentration is relatively small, remaining within the range of −0.79 to −0.80 V_SCE_. In contrast, the corrosion potential in neutral NaCl solutions exhibits a much greater dependence on Cl^−^ concentration.

**Figure 6:**
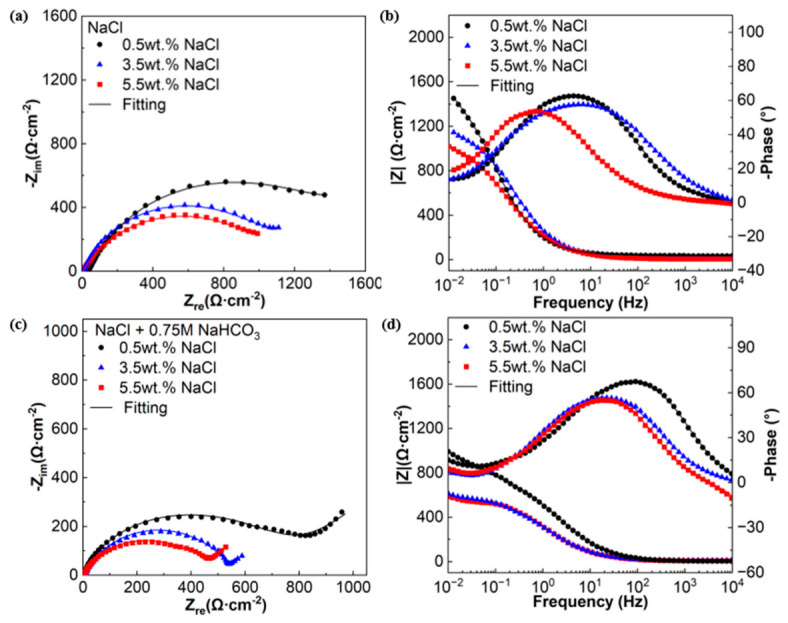
Nyquist plots and Bode plots of pipeline steels in NaCl solutions (**a**,**b**) and NaCl + 0.75 M NaHCO_3_ solutions (**c**,**d**) with different NaCl concentrations.

Figure 6 presents the electrochemical impedance spectroscopy (EIS) results of pipeline steel in mixed NaCl + NaHCO_3_ solutions with different NaCl concentrations, including the Nyquist and Bode plots. The solid lines represent the fitted curves obtained using the corresponding equivalent electrical circuits. Under the same solution environment, the Nyquist plots exhibit similar characteristics. In NaCl solutions, the Nyquist plots display depressed capacitive semicircles. In contrast, for the NaCl + NaHCO_3_ solutions, the high-frequency region consists of a capacitive semicircle, while the low-frequency region exhibits a Warburg-type linear tail, indicating that the reaction mechanism gradually transitions from electrochemical activity control to diffusion control at lower frequencies. The capacitive arc is associated with the response of the interfacial double layer, and its radius is generally correlated with the corrosion resistance of the material. In general, a larger capacitive arc radius corresponds to a lower corrosion rate and superior corrosion resistance [21]. As the NaCl concentration increases, the radius of the capacitive arc progressively decreases for all specimens. This trend is further confirmed by the corresponding Bode plots. Under the same solution conditions, specimens exposed to higher Cl^−^ concentrations exhibit lower impedance modulus values in the low-frequency region and smaller maximum phase angles, indicating a deterioration in corrosion resistance. These observations are consistent with the polarization results.

**Figure 7:**
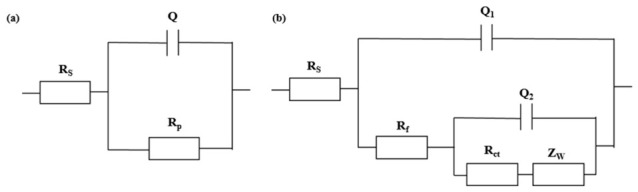
Electrical equivalent circuit for simulating the impedance response of the pipeline steel in NaCl solutions (**a**) and NaCl + 0.75 M NaHCO_3_ solutions (**b**) with different NaCl concentrations.

The Bode plots obtained in the same solution environment exhibit similar impedance and phase-angle responses, suggesting comparable electrochemical behaviors. Accordingly, the equivalent circuits used to fit the EIS data of specimens in NaCl solutions and NaCl + NaHCO_3_ solutions are shown in Figure 7a and Figure 7b, respectively [22]. In NaCl solutions, the Nyquist plots show a single capacitive arc over the entire frequency range, indicating that the corrosion process is dominated by active dissolution. Accordingly, the equivalent circuit shown in Figure 7a was employed for fitting. In this circuit, *R_s_* represents the solution resistance, *Q* is a constant phase element (CPE) accounting for dispersion effects arising from surface roughness and compositional heterogeneity, and *R* represents the resistance associated with charge transfer and anodic dissolution. In NaCl + NaHCO_3_ solutions, a relatively stable protective oxide film forms on the specimen surface; therefore, the equivalent circuit shown in Figure 7b was used. In this circuit, *R_s_* corresponds to the solution resistance between the working electrode and the reference electrode. The parallel combination of *Q_f_* and *R*_f_ represents the oxide film formed on the pipeline steel surface. The parallel combination of *Q_dl_* and *R_ct_* is used to describe the electrochemical charge-transfer process occurring at the metal/electrolyte interface, where *Q_dl_* corresponds to the non-ideal double-layer capacitance, and *R_ct_* represents the charge-transfer resistance governing the kinetics of the corrosion reaction. *Z_w_* denotes the Warburg impedance associated with mass transport by diffusion.

The CPE is widely employed to describe frequency dispersion associated with interfacial inhomogeneity, replacing an ideal capacitor to more accurately characterize non-uniform surface behavior [23]. Its impedance can be expressed as [24]:

(1) $$Z_{CPE}=\frac{1}{Q{\left(\omega i\right)}^{n}}$$

where *Q* is the admittance constant, ω is the angular frequency (rad s^−1^), *i* is the imaginary unit (*i*^2^ = −1), and *n* is the CPE exponent ranging from 0 to 1. When *n* = 1, the CPE reduces to an ideal capacitor; when 0.5 < *n* < 1, it indicates a distribution of relaxation times in the frequency domain; and when n approaches 0.5, diffusion-related processes become significant, resembling Warburg-type impedance behavior [25,26].

The fitted parameters are summarized in Table 3 and Table 4, and the *χ*^2^ values of all EIS fittings were lower than 10^−3^, indicating good fitting quality and reliability of the equivalent circuit model. Variations in surface condition are reflected in changes in the polarization resistance (*R_p_*), which is inversely proportional to the corrosion rate [27]. The overall *R_p_* can be approximated as the sum of *R_f_* and *R_ct_* under the assumption that both processes contribute additively to the total impedance response. A larger *R_f_* value indicates the formation of a dense and protective oxide film, whereas a higher *R_ct_* corresponds to greater resistance to charge transfer across the metal/solution interface and thus slower corrosion kinetics. As shown in Table 3, the *R_p_* decreases from 1623 to 1095 Ω·cm^−2^ with increasing Cl^−^ concentration, indicating a progressive deterioration in the corrosion resistance of the pipeline steel. According to Table 4, the charge-transfer resistance *R_ct_* is substantially higher than the film resistance *R_f_*, suggesting that the corrosion process is predominantly governed by the charge-transfer step. Although a surface oxide film capable of maintaining a stable current density can form on the pipeline steel under alkaline conditions with low Cl^−^ concentrations, the film is relatively porous and provides only limited protection against corrosion. As the Cl^−^ concentration increases from 0.5 to 5.5 wt.%, the *R_f_* decreases markedly from 77.42 to 8.11 Ω·cm^−2^, indicating a substantial decline in the protective ability of the oxide film, which suggests increased film defects and reduced compactness.

Previous studies have shown that ${HCO}_{3}^{-}$ promotes the formation of protective oxide films on pipeline steel, whereas Cl^−^ facilitates film destabilization and anodic dissolution [28,29]. Therefore, the corrosion behavior in NaCl + NaHCO_3_ mixed solutions is governed by the competing effects of ${HCO}_{3}^{-}$-assisted film formation and Cl^−^-induced film degradation [4]. At lower Cl^−^ concentrations, ${HCO}_{3}^{-}$ is preferentially adsorbed onto the surface of the pipeline steel, promoting the formation of an oxide film that protects the steel substrate. As the Cl^−^ concentration increases, Cl^−^ preferentially reacts at the steel surface, leading to the breakdown of the oxide film and causing the specimen to enter an active dissolution state. This behavior indicates that the corrosion process gradually becomes dominated by activation-controlled dissolution as the Cl^−^ concentration increases. However, excessively high concentrations of ${HCO}_{3}^{-}$ can increase the conductivity of the solution and enhance the cathodic oxygen reduction reaction [30]. Therefore, the *R_p_* values of the specimens in the NaCl + 0.75 M NaHCO_3_ mixed solutions are lower than those of the specimens in NaCl solutions with the same Cl^−^ concentration.

**Figure 8:**
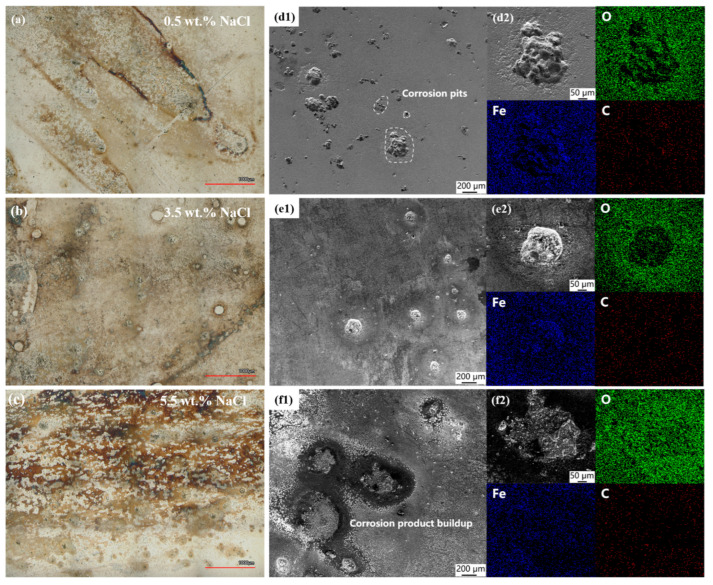
Corrosion morphologies of pipeline steel after potentiodynamic polarization in different solutions: (**a**–**c**) Laser confocal microscopy in 0.5, 3.5, and 5.5 wt.% NaCl; (**d1**,**e1**,**f1**) SEM images in 0.5, 3.5, and 5.5 wt.% NaCl with 0.75 M NaHCO_3_; (**d2**,**e2**,**f2**) Corresponding EDS elemental maps (O, Fe, and C).

**Figure 9:**
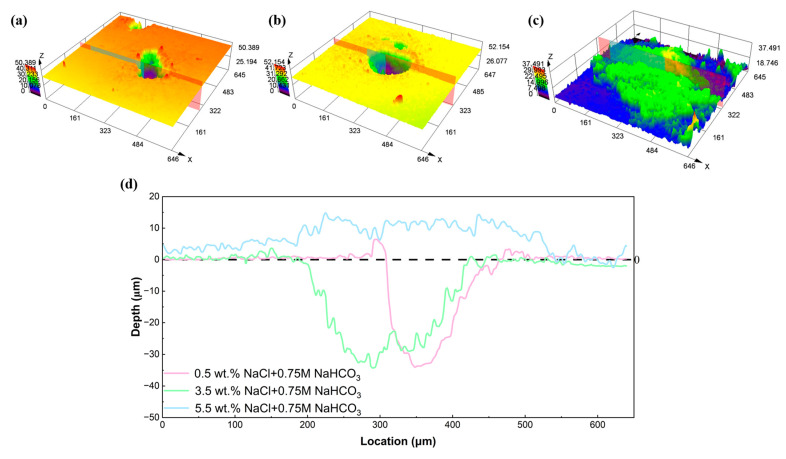
Corrosion morphologies of pipeline steel specimens after potentiodynamic polarization in NaCl + 0.75 M NaHCO_3_ solutions with varying NaCl concentrations: (**a**–**c**) 3D corrosion morphology; (**d**) comparison of cross-sectional depth profiles.

The corrosion morphologies of the pipeline steel specimens after potentiodynamic polarization were analyzed. As shown in Figure 8, all specimens in the NaCl solutions exhibited uniform corrosion. With increasing NaCl concentration, the surface coverage of corrosion products gradually increased. Among all test conditions, the specimen exposed to the 5.5 wt.% NaCl solution experienced the most severe corrosion. In contrast, the specimen in the 0.5 wt.% NaCl solution exhibited a relatively lower degree of corrosion. In the NaCl + 0.75 M NaHCO_3_ mixed solutions, when the NaCl concentration was 0.5 wt.%, localized corrosion was observed on the surface of the pipeline steel, with relatively small corrosion pits of approximately 100 μm in diameter and about 30 μm in depth (Figure 9). When the NaCl concentration increased to 3.5 wt.%, the diameter of the corrosion pits further increased to nearly 200 μm. As the NaCl concentration increased to 5.5 wt.%, the surface of the pipeline steel exhibited uniform corrosion, with no obvious corrosion pits observed, and the surface was covered with corrosion products. This morphological transition from localized corrosion at low Cl^−^ concentration to uniform corrosion at high Cl^−^ concentration further supports the previously discussed mechanism involving the competition between the ${HCO}_{3}^{-}$-assisted film-forming effect and the Cl^−^-induced film-destabilizing effect, with the corrosion process gradually shifting toward Cl^−^-dominated active dissolution as the Cl^−^ concentration increases.

### 3.3. Surface Oxide Film Analysis

Considering that the anodic reaction of pipeline steel exhibits a stable current region in alkaline environments with low Cl^−^ concentrations, a relatively stable oxide film can form on the steel surface. This oxide film can significantly affect the corrosion behavior of pipeline steel in alkaline chloride-containing environments [31]. Therefore, to further evaluate the corrosion resistance of pipeline steel in an alkaline environment with low chloride concentration, Mott–Schottky analysis was performed to characterize the semiconducting properties of the oxide film. According to Mott–Schottky theory, the space charge capacitance of an n-type semiconductor electrode can be expressed by Equation (2), while that of a p-type semiconductor is described by Equation (3) [32]:

(2) $$\frac{1}{C^{2}}=\frac{2}{\varepsilon \varepsilon_{0}eN_{D}}(E-E_{fb}-\frac{kT}{e})$$

(3) $$\frac{1}{C^{2}}=-\frac{2}{\varepsilon \varepsilon_{0}eN_{A}}(E-E_{fb}-\frac{kT}{e})$$

In these equations, *ε* represents the dielectric constant of the oxide film at room temperature, which is taken as 15.6 in this study [33]; *ε*_0_ is the vacuum permittivity (8.85419 × 10^−14^ F·cm^−1^); *N_D_* and *N_A_* denote the donor and acceptor densities, respectively; *e* is the elementary charge (1.6 × 10^−19^ C); *E_FB_* is the flatband potential; *k* is the Boltzmann constant (1.38 × 10^−23^ J·K^−1^); and *T* is the absolute temperature (K). According to the above equations, a larger absolute value of the slope corresponds to a lower carrier density in the film. Therefore, the electrochemical behavior of the metal can be inferred from the semiconductor type and the carrier concentration of the oxide film.

**Figure 10:**
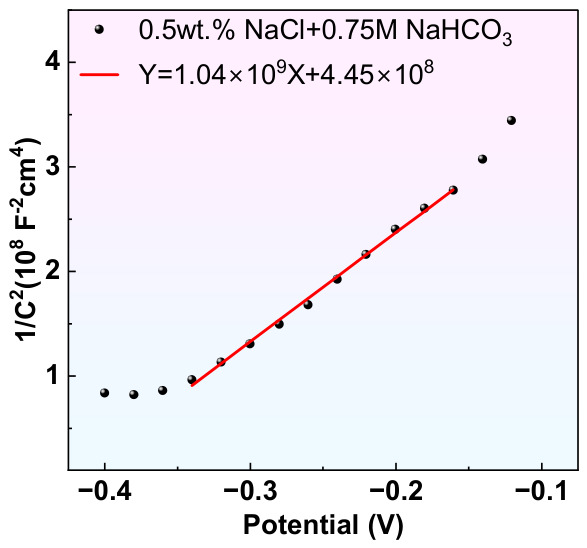
Mott–Schottky plots of the oxide film formed on pipeline steels after potentiostatic polarization in 0.5 wt.% NaCl + NaHCO_3_ solution.

Figure 10 shows the Mott–Schottky plots of pipeline steel in the 0.5 wt.% NaCl + 0.75 M NaHCO_3_ solution. The curve exhibits a good linear relationship within the potential range from −0.35 to −0.15 V_SCE_, with a fitting coefficient *R*^2^ higher than 0.99. The curves exhibit predominantly linear regions with positive slopes, indicating that the corrosion product films formed on the surfaces of the specimens possess n-type semiconducting characteristics. This behavior is associated with the formation of oxide films composed of iron oxides, in which the defects may exist in the form of Fe^2+^ ions or oxygen vacancies, both of which can act as donor states [34]. According to Equation (2), the donor density of the oxide film formed on the pipeline steel in the 0.5 wt.% NaCl + 0.75 M NaHCO_3_ solution was calculated to be 8.69 × 10^21^ cm^−3^. This value is comparable in order of magnitude to those reported in previous studies. However, compared with the oxide films formed on pipeline steels in NaHCO_3_ solutions reported in the literature, the *N_D_* of the film formed in the NaCl + NaHCO_3_ mixed solution is approximately eight times higher than that of the film formed in the chloride-free NaHCO_3_ solution. A higher carrier density generally indicates a less stable oxide-film structure with a higher concentration of defects. This suggests that, under the alkaline low-chloride condition, the oxide film formed on the pipeline steel surface is a defective surface film with limited protectiveness rather than an ideal compact film [35,36].

**Figure 11:**
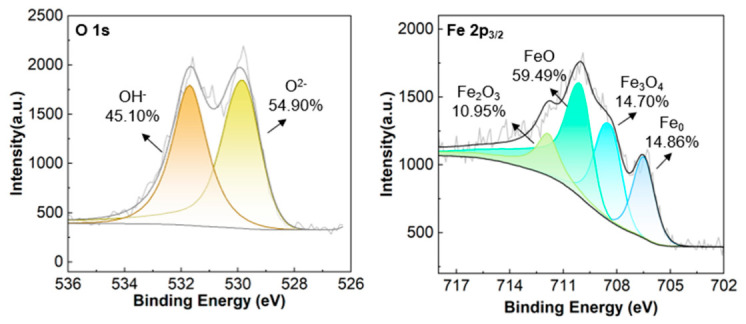
The detailed XPS spectra of Fe 2p_3/2_, O 1s of the oxide film formed on pipeline steel after polarization in 0.5 wt.% NaCl + 0.75 M NaHCO_3_ solution.

For further analysis of the oxide film characteristics, the oxide film formed on the pipeline steel in the 0.5 wt.% NaCl + 0.75 M NaHCO_3_ solution was characterized by X-ray photoelectron spectroscopy (XPS), and the corresponding spectra are shown in Figure 11. High-resolution spectra of Fe 2p_3/2_ and O 1s were obtained. The main constituents of the oxide film include OH^−^, O^2−^, Fe (II), and Fe (III). According to the literature [37], the O 1s spectrum was deconvoluted into a peak at 529.85 eV corresponding to O^2−^ and a peak at 531.70 eV corresponding to OH^−^. The Fe 2p_3/2_ spectrum was deconvoluted into four peaks: one peak located at 706.7 eV corresponding to Fe^0^, and three peaks located at 708.3, 709.4, and 711.0 eV, corresponding to Fe_3_O_4_, FeO, and Fe_2_O_3_, respectively. These results indicate that the oxide film mainly consists of Fe^2+^- and Fe^3+^-related oxides, which is consistent with the Mott–Schottky results discussed above.

Previous studies have shown that the oxide film formed on pipeline steel in alkaline chloride-containing environments typically consists of an Fe^2+^-rich inner layer, an Fe_3_O_4_-rich intermediate layer, and an outer layer enriched in Fe^3+^ oxides/hydroxides [38]. In the 0.5 wt.% NaCl + 0.75 M NaHCO_3_ solution, Fe^2+^-related oxide species account for a relatively high fraction in the oxide film formed on the pipeline steel surface. It has been reported that the formation of Fe^3+^ oxides in the outer oxide layer essentially involves the oxidation of Fe^2+^ to Fe^3+^. The relatively high concentration of ${HCO}_{3}^{-}$ in the solution adsorbs at local defects within the oxide film and inhibits the migration of dissolved oxygen toward oxygen vacancies. Owing to the insufficient oxygen supply, the formation of Fe^3+^ oxides in the oxide film is suppressed. Meanwhile, a large amount of unreacted Fe^2+^ is retained in the inner layer of the oxide film, resulting in an increased content of Fe^2+^-related oxide species within the oxide film [39].

Based on the above results, it can be concluded that the anodic behavior of pipeline steel is strongly influenced by the solution environment and is closely related to the solution pH, ${HCO}_{3}^{-}$ concentration, and Cl^−^ concentration. In pure NaCl solutions, the primary anodic reaction is:

(4) $$Fe\to {Fe}^{2+}+2e^{-}$$

In NaCl + 0.75 M NaHCO_3_ mixed solutions, the addition of a high concentration of ${HCO}_{3}^{-}$ favors the formation of oxides/hydroxides on the pipeline steel surface, thereby reducing the anodic dissolution rate. This corresponds to the anodic current peak observed at relatively low potentials in the polarization curve. The possible reactions are as follows [40]:

(5) $$Fe+2H_{2}O\to Fe{\left(OH\right)}_{2}+2H^{+}+2e^{-}$$

(6) $$Fe+{HCO}_{3}^{-}\to Fe{CO}_{3}+H^{+}+e^{-}$$

In the mixed solution, Fe(OH)_2_ may form on the pipeline steel surface during the initial stage of corrosion, while Fe^2+^ may also interact locally with carbonate species to generate FeCO_3_-related intermediate species under favorable conditions. In an alkaline environment, as the corrosion reaction proceeds, carbonate-related Fe species may further transform into Fe(OH)_2_ or Fe(OH)_3_ and remain attached to the pipeline steel surface [41].

When the Cl^−^ concentration is low, a stable current-density region appears as the potential gradually increases, indicating the formation of a protective oxide film on the pipeline steel surface. The possible reactions are as follows [40]:

(7) $$Fe+2Fe{\left(OH\right)}_{2}+{4OH}^{-}\to {Fe}_{3}O_{4}+4H_{2}O+4e^{-}$$

(8) $$2{Fe}_{3}O_{4}+H_{2}O\to {3Fe}_{2}O_{3}+2H^{+}+2e^{-}$$

When the Cl^−^ concentration was further increased to 3.5 and 5.5 wt.%, *i_corr_* increased, whereas *β_a_* and *R_f_* decreased, indicating a substantial acceleration of the corrosion rate. The deterioration in corrosion resistance is mainly associated with accelerated anodic dissolution kinetics. Previous studies have shown that Cl^−^ preferentially interacts with structural defects in the oxide film at relatively low chloride concentrations, resulting in localized film breakdown while the majority of the surface film remains intact. As the Cl^−^ concentration increases, progressive film destabilization exposes an increasing number of electrochemically active sites, thereby facilitating Fe dissolution and accelerating the anodic dissolution kinetics, as evidenced by the decreases in *β_a_* and *R_f_*. Consequently, the corrosion mode changes from a localized attack to a more extensive uniform dissolution [42]. For oxide films formed on pipeline steel in alkaline environments, Fe^2+^ oxide species have been reported to protect the metal substrate, whereas the Fe^3+^-rich oxide/hydroxide outer layer is relatively loose and less effective in blocking the transport of aggressive ions [43]. It has also been reported that Cl^−^ can promote the degradation of defective oxide films by enhancing defect generation and ionic transport within the Fe^3+^-rich oxide/hydroxide outer layer, which may further affect the stability of the protective Fe^2+^ oxide inner layer and promote its conversion into Fe^3+^ oxide/hydroxide species [44]. This process is consistent with the changes in the anodic polarization curves, the decrease in *R_f_*, and the observed transition in corrosion morphology from localized corrosion to more widespread corrosion.

## 4. Conclusions

Based on the systematic investigation of corrosion behavior, electrochemical behavior, oxide-film characteristics, and surface morphology of pipeline steel in NaCl + 0.75 M NaHCO_3_ solutions with different Cl^−^ concentrations, the following conclusions can be drawn:

(1) The corrosion evolution of pipeline steel under high-bicarbonate alkaline conditions was strongly influenced by chloride concentration variation. Increasing Cl^−^ concentration resulted in increased corrosion current density and decreased polarization resistance and oxide-film resistance, indicating the progressive deterioration of corrosion resistance. The stable current-density region observed at low Cl^−^ concentrations gradually disappeared with increasing chloride concentration, demonstrating the transition from a relatively stable oxide-film-controlled state to an active corrosion state. This evolution was related to the different effects of ${HCO}_{3}^{-}$ and Cl^−^ on oxide-film stability under alkaline conditions.
(2) The oxide film formed on pipeline steel in 0.5 wt.% NaCl + 0.75 M NaHCO_3_ solution exhibited n-type semiconductor characteristics, with a donor density of 8.69 × 10^21^ cm^−3^. XPS analysis revealed that the oxide film mainly consisted of iron oxides, including FeO, Fe_3_O_4,_ and Fe_2_O_3_, with Fe^2+^-containing oxide species as the dominant components. The results provide experimental evidence for the relationship between oxide-film composition, semiconductor properties, and corrosion resistance under chloride-containing alkaline conditions.

## Figures and Tables

**Table 1:** The elemental composition of the experimental materials [17].

Element (wt.%)	C	Si	Mn	P	S	Cr	Ni	Fe
Sample	0.17–0.23	0.17–0.37	0.35–0.65	≤0.035	≤0.035	≤0.25	≤0.30	Bal.

**Table 2:** The electrochemical parameters derived from polarization curves in Figure 5.

Solution	*E_corr_* (V_SCE_)	*i_corr_* (10^−5^ A·cm^−2^)	*i_p_* (10^−4^ A·cm^−2^)
0.5 wt.% NaCl	−0.72	1.08	―
3.5 wt.% NaCl	−0.77	1.73	―
5.5 wt.% NaCl	−0.81	2.54	―
0.5 wt.% NaCl +0.75 M NaHCO_3_	−0.79	1.67	1.05
3.5 wt.% NaCl +0.75 M NaHCO_3_	−0.79	1.83	―
5.5 wt.% NaCl +0.75 M NaHCO_3_	−0.80	3.68	―

**Table 3:** Fitting results of measured EIS data in NaCl solutions with different NaCl concentrations.

Solution	*R*_s_ (Ω·cm^−2^)	*Q* (10^−4^ Ω^−1^s^n^cm^−2^)	*n*	*R*_p_ (Ω·cm^−2^)
0.5 wt.% NaCl	35.34	8.83	0.74	1623
3.5 wt.% NaCl	10.21	13.41	0.75	1189
5.5 wt.% NaCl	5.21	15.18	0.70	1095

**Table 4:** Fitting results of measured EIS data in NaCl + 0.75 M NaHCO_3_ solutions with different NaCl concentrations.

Solution	*R_s_* (Ω·cm^−2^)	*Q*_1_ (10^−5^ Ω^−1^s^n^cm^−2^)	*n*_1_	*R_f_* (Ω·cm^−2^)	*Q*_2_ (10^−4^ Ω^−1^s^n^cm^−2^)	*n*_2_	*R_ct_* (10^2^ Ω·cm^−2^)	*W* (10^−2^ Ω·s^−1/2^)
0.5 wt.% NaCl + 0.75 M NaHCO_3_	7.7	3.58	0.98	77.42	2.78	0.61	7.81	5.33
3.5 wt.% NaCl + 0.75 M NaHCO_3_	5.12	7.53	0.96	18.68	4.81	0.7	5.92	3.28
5.5 wt.% NaCl + 0.75 M NaHCO_3_	3.83	14.3	0.85	8.11	5.70	0.54	3.43	1.28

## Data Availability

The original contributions presented in this study are included in the article. Further inquiries can be directed to the corresponding author.
